# Making a living and zoonotic disease risk management in coloured broiler poultry farms in Northern Viet Nam

**DOI:** 10.1007/s10460-024-10696-8

**Published:** 2025-01-23

**Authors:** Eve Houghton, Khue Thi Minh Nguyen, Ivo Syndicus, Dien Thi Nguyen

**Affiliations:** 1 The Royal Veterinary College, University of London, Hawkshead Lane, North Mymms, Hatfield, Hertfordshire UK; 2 Viet Nam National University of Agriculture, Trâu Quỳ, Gia Lâm, Hanoi, Viet Nam

**Keywords:** Poultry, Viet Nam, Biosecurity, Livelihoods, Zoonoses

## Abstract

This paper asks what influences farmers’ adherence to national and international zoonotic disease intervention efforts and argues that development and promotion of biosecurity interventions must take into account the economic and social context informing how livestock sectors operate and how those who work in them are making a living. Specifically, we explore how poultry farms in Viet Nam are managed amidst global efforts to combat disease and national ambitions to sustain growth. The growth of Viet Nam’s livestock sector has been accompanied by a range of disease outbreaks that have caused the deaths of animals and humans, threatened businesses, and led to the introduction and ongoing revisions to biosecurity efforts. Despite a strict national (and international) agenda focusing on disease control through biosecurity strategies, on farms disease management is implemented in various ways and to varying degrees. Based on fieldwork in three provinces of Northern Viet Nam and in-depth interviews with actors working on farms and across the commercial poultry sector, we reflect on social, financial and political factors shaping the country’s biosecurity narratives and discuss key practices farming households engage in that influence their disease management efforts. Our findings reveal that strict adherence to biosecurity guidelines is often practically unfeasible for commercial poultry farming households to implement where zoonotic diseases are not a concern related to bird and human health so much as a potential risk to a household’s living, that exists among a range of diverse opportunities and uncertainties shaping farming operations in Viet Nam’s changing livestock sector.

## Introduction

The poultry sector is just one of the livestock sectors that has seen dramatic growth in recent decades, particularly in South and Southeast Asia (Mottet and Tempio 2017). In Viet Nam, while backyard production is still practiced in more than seven million households, a steady increase in the number and size of poultry farms around the country has seen the sector grow quickly and towards more intensified commercial production since the 1990s (Delabouglise et al. 2020). This has resulted, as of 2020, in the production of over one million tons of poultry meat per year (FAOSTAT 2022). At the same time, a range of diseases with zoonotic potential are already endemic among poultry populations in Viet Nam, including Newcastle disease and avian influenza. The growth of Viet Nam’s livestock sector has been accompanied by periodic outbreaks of these diseases, killing animals, disrupting economies, and in some instance also harming the human population. For example, since 2003, 129 cases of avian influenza (H5N1) have been identified in humans in Viet Nam, leading to 65 deaths (World Health Organization 2024). The ongoing struggle to prevent and respond to diseases such as avian influenza that can quickly spread across borders, means zoonotic disease prevention and oversight has become an international effort with countries collaborating on shared strategies to combat disease risks and limit the impact of outbreaks when they do occur (Porter 2013). In Viet Nam, this includes membership in an international One Health Network with other Association of Southeast Asian Nation (ASEAN) countries, among others (ASEAN 2023). While any agreements reached in these international forums extend beyond a nation’s border in conception, in practice implementation often remains country specific and One Health and disease prevention become entwined with larger national agendas. In Viet Nam, this includes an agenda of livestock sector growth and extension of international trade, including the export of poultry. In an effort to meet these aims while also limiting the risk of disease, the government has restricted slaughter in certain wet markets in favour of automated slaughterhouses and introduced policies encouraging land consolidation and concentration of production, moving away from household production to create an attractive environment for private companies to operate at large scale (Vu et al. 2024; Hanh et al. 2007; To et al. 2019). Thus, biosecurity is woven into national land and food system transformations through narratives with a high-tech, industrial agricultural vision at their heart.

While these international commitments and sectoral ambitions are impacting poultry production and distribution, interventions pitched as being both good for biosecurity and the economy have so far failed to address interspecies interconnections in ways that consistently prevent infection. The cause of this failure, Porter argues, is that One Health governance ‘fails to properly account for the conditions under which farmers and fowl actually live’, meaning even the most well-meaning intervention efforts can fail to be successfully implemented (Porter 2019, p. 14). In Viet Nam, this may well be the case, as while policies are looking forward to a particular kind of industrialised and biosecure future, in the North of the country, coloured chicken^1^ and wet markets remain a prominent source of poultry meat and the sector is still predominantly made up of farms that are established and run by independent farmers who take direct responsibility for work on the farm themselves or as a household. As such, farmers remain central to the decision making on farms that have consequences both for their livelihoods (within their capacity to influence this) and disease management. In an effort to understand the interplay between national biosecurity efforts and on farm disease risk management, this paper explores poultry farming practices as they relate to both Viet Nam’s biosecurity narratives and farming livelihoods. In exploring this dynamic, we contribute to livelihoods research that considers local livelihoods as embedded in, and in thrall to, power relations at higher levels (Suhardiman and Rigg 2021; de Haan and Zoomers 2005). Building upon the Sustainable Livelihoods Approach (SLA) and later framework that became popular among academics and development agencies in 1990s (Department for International Development 1999; Chambers and Conway 1992; Scoones 1998; Natarajan et al. 2022) and in which assets and the ability to respond to shocks were central, de Haan and Zoomers were part of a move to acknowledge how power relations and asymmetries between actors can impact livelihoods—aspects that the SLA was lacking and rightly received criticism for. Their work, along with the likes of Scoones (2009) sought to better acknowledge the influence of politics, globalisation and historical processes (such as colonialism or structural adjustment policies) upon livelihoods—challenging the focus (and in some cases responsibility) often placed on local actors in one place in the present. This has led to works that have sought to expand the framework and explore ‘livelihood making’ as ‘pathways’ through time, space, perspectives, and relations (de Haan and Zoomers 2005; Vicol 2018; de Jong 2013).

This lens allows us to look at the livelihoods of poultry farmers and think about how they are situated within broader historical and political contexts informing the choices (and limitations to options available) made on farms. Drawing on the work of de Haan and Zoomers, we explore this subject from the position that people do make their own livelihoods, but “not under conditions of their own choosing” (2005, p. 43). Thus, combatting disease in the current context amidst sectoral growth requires disease management strategies that better acknowledge the social, historical and political-economic conditions that allow transmission routes to form (Porter 2019). To address this, the question of how actors currently make a living within commercial poultry production and distribution networks must be explored (Hennesey et al. 2021). By looking at how global health agendas and disease risks are influencing the livelihoods of poultry farmers, this work seeks to contribute to this body of literature. Equally, by looking at how livelihood strategies are influencing global health and disease risks, we also hope to extend upon it.

As a contribution to demonstrate how consideration of livelihoods amidst concerns of zoonotic disease risks can enhance and practically refocus One Health discussions and intervention efforts, this research describes key practices farming households engage in to make a living. This work draws on findings from qualitative interviews and observations on commercial farms with flocks of over 2000 birds. Through description of farmers’ experiences and choices relating to chicken breeds, seasonality, and the farm’s scale of operation, we consider the impact that these decisions have on farming practices. Then, by situating these practices within the broader social and economic context, we reflect on how various opportunities and constraints are shaping farmers’ attempts to make a living. In each case, we consider specific farming practices observed or discussed with participants during fieldwork against transnational health community and national government concerns about zoonotic disease risks associated with poultry farming and the biosecurity measures suggested to address them. In doing so, we demonstrate that there is a disconnect between the biosecurity measures promoted by the Vietnamese government and international health communities, and the disease management strategies implemented on commercial coloured chicken farms in Viet Nam. We argue that this disconnect is evidence of a disparity between the sanitised and standardised farm that biosecurity measures inherently promote and the adaptable and heterogenous approaches taken in Northern Viet Nam’s coloured chicken farms that are contributing to farming households’ livelihoods. We further suggest that development and promotion of biosecurity interventions to aid zoonotic disease risk mitigation strategies in different countries must take into account the economic and social context informing how farms operate and how those who run them make a living. In addition, location specific research should be used to reflect upon how global health narratives are shaping the kinds of interventions being implemented and their potential consequences within a given context.

Following on from an overview of methods, we introduce the concept of biosecurity as it relates to the poultry sector in Viet Nam. Then, we discuss the opportunities actors associate with poultry farming that shape how they establish their farms, as these preliminary decisions and motivations can ultimately inform how the farm is managed, with potential consequences for biosecurity implementation. The fourth section of this paper explores how coloured chicken farming has emerged and remained in Viet Nam as part of a viable livelihood strategy, and describes the decisions actors make surrounding breeds, batch management and seasonality of production that inform practices on farms. We conclude by exploring mixed farming and *gà dù*^2^—two specific practices some farmers employ to enhance their living and the biosecurity challenges that can be associated with each. Our findings reveal that strict adherence to biosecurity guidelines is often practically unfeasible for commercial poultry farming households to implement where zoonotic diseases are not a concern related to bird and human health so much as a potential risk to a household’s living, that exists among a range of diverse opportunities and uncertainties shaping farming operations in Viet Nam’s changing sector.

## Methods

Drawing from a dataset of 80 interviews with a range of actors associated with the breeding, raising, transporting and sale of chickens, this paper is predominantly informed by 16 in-depth, face-to-face, semi-structured interviews conducted with poultry farmers and the corresponding visits to their farms. Some of these interviews were with multiple participants from a single household (*n* = 19: 14 male and 5 female). The fieldwork and interviews were conducted by three researchers who conducted field visits to farms and other field sites individually or occasionally together between June 2021 and February 2023. Researchers used a shared interview guide to provide a framework and key areas of discussion to explore during interviews. This included a range of subjects such as the history of the farm, where inputs were sourced, how birds were sold, experiences of disease outbreaks, and the farm’s income and expenses (among many others). While this guide gave some shape to interviews, the researchers took a semi-structured approach, allowing us to adapt to each participant and respond to emerging topics of interest. During the period of fieldwork, researchers met weekly to discuss interviews as a group.

Our work took place in three provinces of Northern Viet Nam: Hanoi capital, Bac Giang and Hai Duong. These provinces include examples of Viet Nam’s diverse environment, with Bac Giang’s hills differing greatly from Hanoi’s peri-urban centres and Hai Duong’s connection to the Red River Delta. These provinces were selected based on their high concentration of poultry farms and in alignment with other fieldwork being conducted in the UKRI GCRF One Health Poultry Hub—the project within which this research sits. The interviews took place in Vietnamese and were recorded with participants’ informed consent. Field observations took place alongside these interviews.

While we conducted fieldwork on a range of farms that informed our understanding of the sector, this study explores the workings of commercial farms with capacity to raise more than 2000 birds per batch.^3^ Viet Nam’s Ministry of Agriculture and Rural Development (MARD) uses a combination of income and production quantities to classify farms as *trang trại* or “commercial” (Cesaro et al. 2019) and other research varies in their categorisations. Our identification of commercial farms has thus been informed by our connection to the broader GCRF project in which our work sits where any farms of 2000 birds or more are referred to as “large” or “commercial” within the context of Viet Nam (Nguyen et al. 2023). Farms of fewer birds are then conceptualised in various ways based on either scale (small/medium) or farming location or techniques (backyard/traditional/semi-intensive). Sometimes, scale and location are used together to categorise farms, such as in Burgos et al. (2008) where they define backyard flocks as anything up to 50 birds. In this work, we have solely used quantity of birds per batch to inform our definition, and refer to farms of 2000 birds or greater per batch as “large”, and anything less as “small”. In doing so, our analysis has been able to focus on farming households who sell birds into a broader network of traders and markets, that have ramifications for potential disease transmission through transport and wet markets. Here, a household refers to a set of relationships between people occupying or with shared responsibility for the provision of essentials for living, that often revolves around a property or shared space.

The findings of this study are drawn from our work on coloured broiler farms. Coloured chickens refer to the indigenous and hybrid breeds that make up 72% of the total broiler population in Viet Nam (Nguyen et al. 2023). This selection was motivated by the fact that coloured broiler farms are more common than any other type in Viet Nam.

Initial contact with farms was supported through contact with local government officers in the three provinces where research took place. Often, an officer would accompany us on field visits.

Farms included two of Viet Nam’s commercial farming modalities: independent farms and contract farms (Table 1). At their simplest, contract farms are those which receive inputs from companies and sell back mature birds at a guaranteed price, while independent farms use cash or credit arrangements with feed and pharmaceutical dealers acquire the inputs that allow them to raise and sell birds. If opting to receive inputs on credit (or only able to afford them this way), farmers accept a slightly elevated cost, but only having to pay for them once a batch of birds has been sold. Notably, dealers and companies are not the only means of acquiring inputs for farms, with informal providers of certain provisions also available in some areas. For example, some farmers supplement their feed with leftovers from school canteens.

Following the completion of interviews, recordings were transcribed and translated into English. Researchers took a purposive approach to analysis based on the research focus. Some specific codes were defined prior to analysis. These codes were not finite, and many additional codes and reframing of topics took place inductively during the coding process and group discussions among researchers. Researchers used MAXQDA software to manage, share, code and analyse interview transcripts and fieldnotes.

The interpretation of data following the coding process developed through collaborative writing and discussion across the research team. Initially, this involved discussions to explore whether researchers had a shared understanding of the transcripts and field experiences, what stood out to them and where opinions differed. Patterns and points of divergence were identified and discussed in light of relevant literature and the study’s focus on what any finding could mean for disease risk management came together to inform the aspects of the data that were ultimately chosen for more extensive examination. Patterns that emerged that seemed entirely unique or had subtle differences from other contexts that operate in similar ways (for example with mixed farming and *gà dù*) were identified as appropriate examples to explore in detail in light of their potential influence on disease risk management and contributing to broader literature on food systems, intensification and One Health.

**Table 1:** Farm types visited based on farming modality, chicken breed, and quantity per batch

Type of chicken	Independent farm		Contract farm		Total
	Small scale farm (< 2000 birds/batch)	Large scale farm (> 2000 birds/batch)	Small scale farm (< 2000 birds/batch)	Large scale farm (> 2000 birds/batch)	
Coloured broiler	8	13	1	3	25
White broiler	1	4	0	1	6
Total	9	17	1	4	31

This study focuses on findings relating to the 16 interviews conducted with large scale coloured poultry farms, while drawing additional sectoral knowledge from all the farm visits

### Biosecurity narratives and their application in Viet Nam

Before discussing how commercial poultry farms are run in Viet Nam and what that could mean for efforts to control zoonotic disease outbreaks, it is important to understand the dominant narratives that are shaping what is expected of poultry farms and people who manage them. Specifically, we explore how one prominent narrative in international health and development efforts has influenced policy in Viet Nam: biosecurity.

Disease outbreaks among livestock have had consequences for health and economies at local and global scales in recent decades. The defence against those outbreaks that pose a risk to humans and other animals are now regularly represented by the term ‘biosecurity’^4^. Narratives that situate disease in space have emerged at the forefront of biosecurity strategies, and many of the preventative and responsive disease risk management measures that have been promoted reflect this (Hinchliffe et al. 2013; Mather and Marshall 2011a). As Hinchliffe et al. (2013) discuss, biosecurity has appeared as a rebranding and extension of disease management strategies that have been around for hundreds of years, hinging on borders that distinguish healthy from unhealthy conceptually and physically through acts of separation, surveillance and operational integration.

With the threat of some diseases placed firmly (and in some cases literally) at the farm door, efforts to combat disease risks using a range of biosecurity measures have had ramifications for farmers and animals across the world. Depending on the location and perceived level of risk among policy makers or contracting companies, biosecurity might include anything from the introduction of vaccinations, to restricting the movement of people and infected animals, or in some cases the enforced culling of livestock (Degeling et al. 2016). One event that is responsible for dramatic changes in vaccination policies and a proliferation of farm-based biosecurity interventions globally, took place in 2003, when Viet Nam was one of the first countries to report an outbreak of the H5N1 strain of High Pathogenic Avian Influenza (HPAI). In Viet Nam, the outbreak led to the pre-emptive culling of more than 44 million birds and the deaths of more than 50 people (The World Bank 2014). Since then, other avian influenza outbreaks have also threatened the country’s poultry, including the H7N9 outbreak in neighbouring China in 2014 (Butler 2014). In each case, the government and other independent agencies have worked to improve biosecurity in the poultry sector through strategies such as increasing the number of government vets and improving the country’s diagnostic capacities (Butler 2014). These efforts, which have led to changes in policy, investment and the shape of the sector, have had mixed success. For example, following the outbreak of HPAI in 2003, despite a large amount of financial support from foreign donor countries such as the USA to help combat the disease, Viet Nam did not manage to reduce instances of outbreaks beyond the other countries fighting the problem in Asia (Vu 2009).

Since then, Viet Nam has not wavered in its commitment to policies and investments in biosecurity that have direct consequences for those working and seeking to consume livestock. A core part of Viet Nam’s biosecurity narrative is that, when done right, it can prevent disease and make food safe while sustaining growth—something the government has remained committed to across the poultry sector as they seek to establish the country as a major exporter. This commitment is evident in their national policy that has sought to encourage producers across food sectors (not just poultry) to start meeting international food safety standards (Duteurtre et al. 2022), an emphasis that research has shown to favour large-scale industrial farms and often force consolidation (Fearnley 2020; Dunn 2003). Equally, their commitment to economic growth is seen through land law reforms that, since the Twelfth Party Congress in 2016, have encouraged land consolidation and recognised land concentration under the private sector as a priority (Duteurtre et al. 2022) and “the engine of modern agricultural production” (To et al. 2019). Thus, these reforms facilitated the repackaging of land into larger plots and sale to international companies for use in large scale industrial, agricultural and livestock production ventures. For example, in 2020 Charoean Pokphand Vietnam (CPV) opened Southeast Asia’s largest chicken breeding and processing facility, primarily to grow Viet Nam’s international export market. With capacity to raise and process 100 million white broiler chickens per year, the facility was opened by the country’s Deputy Prime Minister and (according to CPV’s self-promotional reporting on the occasion) the facility falls within a “Disease Safety Zone” with a “system of barns” that protects birds from disease by adhering to World Organisation for Animal Health standards (CPV Food 2020).

**Figure:**
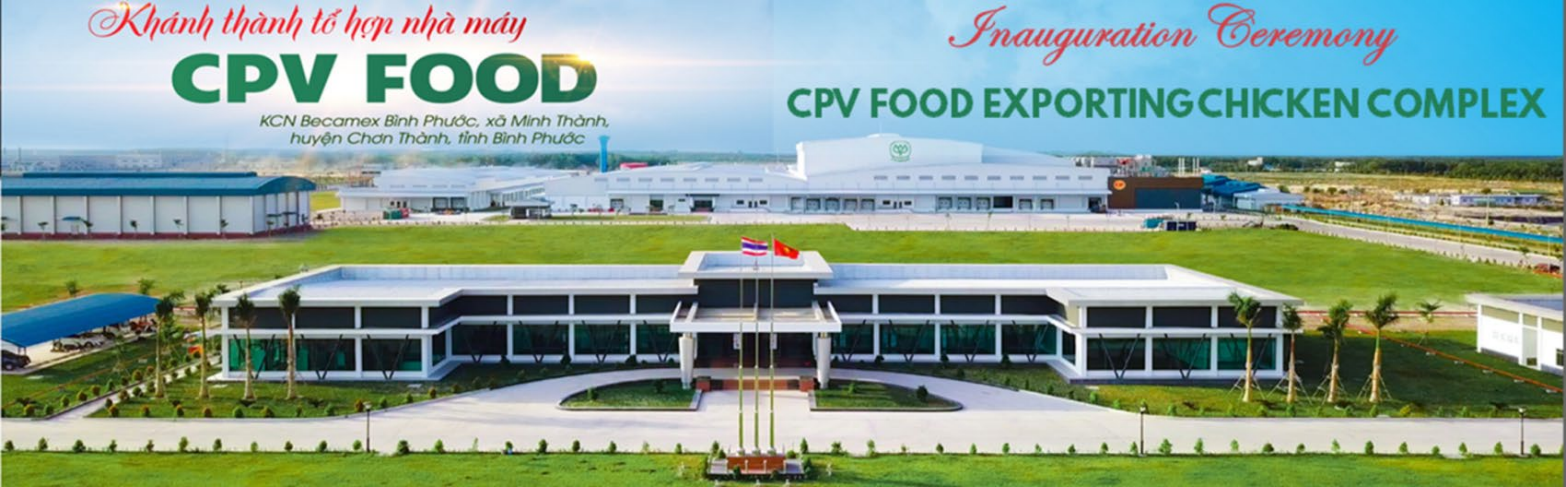
Photo credit: CPV Food via PR Newswire

This dual commitment to both disease prevention and sectoral growth and export have produced biosecurity narratives that promote standardisation across systems and places. As Hinchliffe et al. describe in relation to operational integration:

‘Regulation and compliance seem to be more complex in conditions of operational and environmental diversity, and dealing with fewer players and having clear reporting lines are often regarded as enhancing biosecurity implementation’ (Hinchliffe et al. 2013).

This agenda is not unique to Viet Nam, with research from around the world revealing national and international disease prevention and growth objectives that implicitly or explicitly prioritise intensification, international relations and export standards (Dixon 2015; Hinchliffe et al. 2021). For example, Mather and Marshall’s work on ostrich farms in South Africa reveals the national priorities following an avian influenza outbreak, in a response that echoes Viet Nam’s following the outbreak of 2003:

For South Africa’s Department of Agriculture, managing the disease decisively and according to international best practice was important in order to re-establish export flows quickly. But following the rules was also about demonstrating the country’s commitment to being a “good citizen” in the global struggle to control emerging infectious diseases (Mather and Marshall 2011b, p. 154).

Thus, national aspirations for growth, trade, disease management and food safety standards are informing policies that impact farming operations, both directly and through regulating the networks they need to access to operate^5^. In Viet Nam, along with a host of vaccine requirements and enhanced veterinary oversight, particularly relating to the country’s many wet markets, much of the emphasis has been placed on industrialisation as a means to make the sector more biosecure and meet international export standards. This is evidenced by the country’s HPAI strategy developed by the Ministry of Agriculture and Rural Development (MARD) in 2006 (and iterated upon since). As Porter explains, policy restrictions shaping access to wet markets and slaughter facilities in urban centres, along with rules that only industrially integrated poultry varieties can be sold there, have had significant knock-on effects on who can access these markets to sell their birds, the types of birds being sold, and the way that farmers have needed to adapt their holdings to fulfil these new requirements. MARD “hoped to see industrialized and semi-industrialized poultry farms account for 80% of domestic poultry production by 2015” (Porter 2019). While they did not reach these numbers, 20% of small commercial operators and household producers did leave the industry in response to market and slaughter restrictions (Porter 2019). For those who remain or have joined the sector in recent years, adaptation has been required to adhere to rules or find new ways to sustain their business.

Around the world, the investment in industrialised agriculture and broader national politics have led to agricultural transitions and disruptions, with various results. Land dispossession, political unrest, restrictive new policies and fluctuating markets have all been responsible for shrinking the number of non-industrial livestock farming operations in different countries (see, for example, Bai et al. 2018; Scoones 2020). Yet, while farms remain challenged in many contexts, it is vital to remember that backyard, small and independent farms remain, and often constitute a large part of local markets and household nutrition alongside growing contract and industrialised operations. Research from China (Fearnley 2020) and Cambodia (Ebata et al. 2020), exemplify different global contexts in which intensification “alters rather than replaces the mixture of production types and practices” undermining the standardisation of food safety and biosecurity efforts that some believe intensification can provide (Hinchliffe et al. 2024: 8). In Viet Nam, it is telling that popular coloured chicken breeds still dominate the local market and are predominantly produced through non-industrial^6^ farming operations.

## Choosing chicken farming

It is important to understand the business of chicken farming within a broader social context that informs its reputation and how farmers enter into it, as this may have ramifications upon the adaptations farmers are willing or able to make relating to guidance for disease prevention.

While policy changes have seen many small-scale producers leave the sector, larger commercial household farms continue or choose to join, drawn by a certain appeal that the sector continues to hold even as the national agenda seeks to limit the role of household poultry farms. In line with the country’s sectoral growth strategy, positive narratives surrounding poultry are circulated in Viet Nam through government initiatives, private company marketing and investments, associated stakeholders (such as dealers selling feed and other inputs associated with poultry) and in many instances, local farming cooperatives and between individuals as well. These sources have helped shape the sector’s reputation among household farmers, with particular positives standing out and encouraging actors to engage with the sector. Specifically, when discussing their perceptions of the poultry sector and why they started to raise poultry, participants highlighted their belief in the sector’s financial dependability, with multiple business opportunities associated with the sale of birds and their waste. Participants also noted the technical simplicity of raising broiler chickens (of either colour), the ease of establishing a farm and the lack of policy restrictions requiring rigid oversight on the farm.

Mr Luu is an example of someone who considered all of these factors when choosing to start raising poultry. A 52-year-old man, Mr Luu explained that he used to only be involved in agriculture—growing rice and grapefruit. Since 2013, his farm has adapted to include aquaculture and poultry, to the point that he is now rearing up to 3000 birds in a batch:

In 2013… the family had some money and realised that raising chickens was profitable, so we wanted to develop broiler farming. [We chose] to raise chickens and not other livestock because broiler farming does not have wastewater, so there is no fear of affecting the environment. Besides, the chicken manure can be collected and sold to growers. Because it has less impact on the environment, it is easier to apply for a permit to raise chickens than to raise pigs. There is no need to make project documents on environmental treatment. [We chose] to raise broiler chickens without choosing to raise laying hens because raising broiler chickens is technically simpler, the payback time is fast, so it is less risky than raising layer chickens and does not require high technology and large investment.’^7^

Mr Luu is not alone in this. The perceived low bar for entry is a core part of the reputation that poultry rearing has in Viet Nam. For some, going ‘back’ to farming is seen as a legitimate and dependable plan if other business or work opportunities are not sustainable. Some participants who had shifted from other sectors into farming, also saw their investment in land as reliable, considering the land to be a dependable asset. While land tenure periods have increased over time, land laws in Viet Nam still mean the government technically retains ownership, selling it with tenure of up to 50 years and a guarantee from the tenant that it will be used for whatever the government has classified it for (Nguyen et al. 2020). Hence, in investing in agricultural land, actors are limited in options when it comes to how they might (legally) opt to use it. In the case of Mr Luu, his description reflects the constraints that he perceives in other farming sectors that he could have chosen to use his agricultural land for, informing his decision to opt for broiler poultry farming over other livestock^8^. Notably he does not associate poultry farming with the needs for high-tech methods or investments, a feeling shared by all the participants we spoke with and that differs significantly from the ambitions the government has for the sector.

Instead, dependability and ease are often the justifications that farmers give for engaging in poultry production. This desire for technically simple farming with relatively quick cycles of reliable income from birds, informs the breed and quantity of birds that people choose to raise, and the farming infrastructures they ultimately invest in to sustain this activity. While this reputation does not reflect what farmers then experience, the reputation associated with the sector may have some influence in how amenable farmers are to efforts relating to disease risk management. For example, while most farmers included vaccinating birds, cleaning houses and sourcing medicine for birds when they show signs of illness as a standard part of running their farm, the desire for simple farming that leaves time or physical space for diverse land use and business operations simultaneously, can limit the uptake of biosecurity interventions at the farm level. Similarly, most of the participants we spoke with run their farms as a household, with each member contributing their labour to the daily running of the farm. The work to upkeep the farm therefore needs to fit within the abilities of the household unit, with some support from neighbours in busy periods such as applying vaccinations to large flocks, rather than needing additional full time or expert labour.

## Decisions shaping farming practice: breed, scale, cycles and seasons

Starting to raise poultry as a commercial endeavour comes with choices farmers must make regarding the breed, cycles, seasonality and scale of their farming operation. These are by no means fixed, and shift and adapt over time based on farming experience and opportunities (or limitations) enabling or forcing farmers to make adaptive decisions. These choices do not exist in isolation from one another, with poultry farming households responding to interconnections between these choices and additional external factors (such as market price) which ultimately informs their livelihood strategies and connect to the relative disease risk or mitigation attempts that play out at the farm level.

Within Viet Nam’s numerous wet markets^9^, live birds of local and coloured hybrid varieties demand a premium price and retain an association with quality that does not carry to white broilers. Customer preference based on breed (or at least appearance of coloured feathers) is substantial enough that it contributes to the reasoning independent farmers give for opting to rear coloured breeds. Indeed, some of the large companies working with contracted farms in Viet Nam also produce coloured breeds, selling them through contracted traders into the wet markets.

Regardless of the intensification that coloured chicken farming has undergone, these birds are still associated by customers with backyard rearing and better meat—a reputation that holds for birds raised by large scale producers. This reputation influences customer perceptions of the meat itself, with many people feeling that coloured birds are more likely to be raised in non-intensive backyard systems and thus be healthier to consume. This reputation combined with the extended raising period needed for coloured broilers to reach ‘maturity’^10^ (70–100 days rather than 35–50) means they are sold for more than white broilers. Farmers explained that they don’t mind raising the birds for longer, because of this higher and more dependable sales price:

Coloured chickens are healthy and easy to raise, easy to sell, and have a higher selling price than white chickens.’^11^

The choice to raise coloured breeds informs how farmers operate. While farms vary, two core patterns emerged shaping how, when and at what scale farmers choose to operate coloured chicken farms which links to their biosecurity implementation: the physical infrastructure of their farm and the season.

The physical infrastructure and land available for use as part of each farm influenced the number of birds farmers chose to raise, as farmers associated the healthy raising of each bird with a specific amount of space. Hence, square footage of each bird house dictated the maximum number of birds they could keep. Typically, farmers described keeping 8–12 coloured birds per square meter of housing, with farmers adapting this figure over time based on their experience and any additional land the birds are given access to. Those engaged in contract farming have some of the calculations for their contract based on the size of their poultry houses and the assumption that no more than 10 birds will be housed per square meter, although in reality the numbers kept did vary.

Beyond the connection between space and health (or in some cases welfare^12^) attributed across chicken farms, the space given to each coloured bird was also associated with their appearance, which is commonly used as a reflection of quality in Vietnamese wet markets. Due to their predominant sale in wet markets, local coloured chicken breeds, such as Ri, Choi and Ho, are all sold to consumers who often see and specifically pick the bird they want to buy before it is slaughtered. Their appearance has a direct impact on how likely they are to get sold and at what price, with large healthy-looking birds selling for far more than *gà xấu* (ugly chicken)^13^. These considerations impact how birds are kept and in some cases, the supplements they receive on farms:

Seven to ten days before selling the chickens, I feed the chickens with stimulants to make their crests red and feathers smooth.’^14^

The desire for coloured birds to appear a certain way and have tough meat, also means many who farm them opt to have some degree of free roaming space for birds. This space is shaped by the land available and is often adjacent to or overlayed upon other agricultural operations. This freedom of movement has a practical goal but is also practiced by many because of the confidence they have that coloured breeds are stronger and less susceptible to disease than white broilers. This reputation has a potential impact upon the disease risks present on farms of different breeds, with those raising closed-housed white broilers likely to keep a greater number of birds, but with relatively less chance of outside incursions. The indoor living and high stocking density can increase stress, which in turn makes birds more susceptible to disease should it strike (Nasr et al. 2021). However, with coloured birds, while their outdoor living and extra space avoids the stresses of confinement, their exposure to wild birds, rodents, and other farming operations means disease risks may become more likely (Salaheen et al. 2015). Thus, in their decision to raise coloured broilers and in seeking to use simple rearing techniques that will simultaneously satisfy the tastes of discerning consumers in wet markets, farming households may reject closed housing models and rigid borders between agricultural operations.

The physical infrastructure of a farm also informs how farmers can manage each batch of birds. Some farmers described operating an ‘all-in all-out’ approach where they intake the full number of chicks they wish to raise and then sell them all in one go once they are mature. This approach is sometimes preferred due to the simplicity of oversight involved and the requirements of contract companies. Following sale, most farmers leave their bird house(s) empty for about two weeks before arranging for a new batch to arrive. Farmers associate this empty shed period with disease management and practical time allowing for the sale of manure and rice husks—a process that clean the sheds and provides additional income.

Alternatively, some independent farmers with multiple bird houses choose to keep different ages of birds in different houses simultaneously. This can involve moving birds between the houses as they grow (with younger birds being kept indoors more and in houses with greater provision for temperature control). If their houses lack a substantial difference that would warrant moving birds between them, then farmers can also opt to stagger production, bringing new chicks into each house at different times, and allowing a more frequent source of income rather than operating an all-in all-out system requiring a higher initial investment and more dependence on the market’s stability at time of sale.

Yes, I raise in the overlapping way. One [new] flock per month. Now there are four generations of chickens. This flock is about to sell out, then I have a new flock preparing to enter…In general, there are chickens sold out every month. That is advantageous, otherwise you can lose. Profit is small, and loss is from a few tens to hundreds of millions due to firstly the price, and secondly an epidemic. In farming, it is not possible to avoid the risks.’^15^

How birds are housed and how quickly houses are refilled after sale has consequences for a household’s income flow and their management of biosecurity. For example, avian influenza viruses can remain viable in chicken manure for up to 20 days depending on the exact subtype and temperature (Lu et al. 2003), thus, for those farmers moving birds between houses, a lack of proper cleaning can easily result in disease transmission within the farm, even if leaving a house vacant for the two-week period that many farmers practice and when birds have freedom to spend most of their time outside of the house itself.

Interconnected with these considerations, farming practices vary depending on the time of year as farmers adapt the quantities of birds they raise in each batch based on how cold, hot, wet or dry they expect it to be. For example, some farmers reported raising more birds in the dryer summer months as birds are cheaper to feed and house with no heating costs. However, this option is only available on those farms able to counter extreme heat or humidity with fans. Those without the means to regulate temperature are faced with the choice of lowering the number of birds raised to protect them from heat stroke or adapting their farm in other cost-effective ways to help cool birds down, such as allowing birds to roam freely in shaded areas or building a pond near bird houses that provides a natural cooling system through sheer proximity. Notably, these ponds can make farms increasingly attractive to wild birds and further complicate biosecurity concerns associated with specific farm layouts and operations.

In winter, farmers adapt in other ways as they associate the cold and wet weather with an increase in the risk of respiratory diseases. Despite this, some farmers increase their production while many cease raising birds entirely. This decision is informed by breed, as local breeds are in high demand during winter and can be sold for a premium in the lead up to Tết^16^, in particular. Thus, farmers may feel the opportunities afforded for chicken sales during this season outweigh any additional risks:

The months next to Tết, there is always higher demand for chicken. Each client can buy up to 10 or 15 birds.’^17^

For those not raising local breeds, the risks associated with the season means they may not raise any birds during winter months. In addition to disease, winter months are associated with a need to buy extra feed to get birds to a mature weight. Farmers also reported more fluctuations in the market during the winter. These factors combined means it costs independent farmers more to raise birds in cooler months while facing greater disease risk:

When I raise the chicken litter I usually avoid January and February of the lunar calendar^18^ because these are the rainy and cold time to keep chickens, which often costs a lot of electricity, leading to higher costs. The risk of chicks dying in this season is often higher than in another.’^19^

Contract farmers more often maintain production across the seasons, altering quantities of birds when temperature changes, but otherwise unimpacted by negative fluctuations in the market as they are guaranteed a set price from the company they work with.

The decisions farmers make to navigate through perceived economic and disease risks means there is an informal disease management strategy at work on most poultry farms. This is often in addition to vaccination programmes, medical interventions and periods of leaving sheds empty between batches—all commonly used interventions promoted by both government and companies to differing degrees. Adapting to perceived risks and opportunities associated with different seasons, and the weights when coloured breeds are considered mature ultimately combine to inform the number of batches farmers produce each year, with three batches being the most common among the farmers we spoke with. The approach farmers take to managing each flock and how they are sold is additionally informed, as we have seen, by the changing market, and physical infrastructure and land farmers have access to. The patterns of practice described in this section, such as the decisions to let flocks live outdoors or for operating a staggered batch rearing cycle, have a direct impact upon the livelihoods of farming household, changing the timing of income, oversight required for birds, or the value of their mature birds. Concerns about disease are not absent from these decisions, with many farmers diligently applying vaccines and leaving sheds vacant between batches. Thus, we see how bird health contributes to farming decisions—the death of a flock was seen as the biggest crisis you could experience on a poultry farm. Yet, this does not lead to farmers investing or implementing strict biosecurity (with changes of boots, use of foot dips, or limitations on visitors to farms, for example). Instead, farmers are selective, opting to use disease management strategies that can work without threatening or disrupting the practices they are otherwise dependent on to enhance or sustain their livelihoods. It is through this practical navigation of perceived options (or constraints) that actors undertake—not always consciously—to sustain their livelihoods, that the disconnect between coloured chicken farming practices and the national biosecurity agenda emerges. To understand how context is informing this disconnect in more detail, the following section explores two key points of divergence from biosecurity standards and what they are felt to provide by the farming households that use them.

## Mixed farming and *gà dù*

### Mixed farming

When researching poultry farming in Viet Nam, one is quick to realize that ‘chicken farms’ and ‘chicken farmers’ are not strictly what those titles imply. Rather, the farms are regularly operated as mixed farming operations, with farming households engaging in a greater network of relationships and income generating activities in concert with their chicken raising. While some of these directly stem from the chickens, such as selling their manure for use on agricultural land, others include activities such as selling rice from their own paddy fields, or going off farm to work in other non-agricultural industries. Thus, the dedicated poultry network in which the decisions and associated practices described in previous sections can be conceptually situated, do not account for the full extent of practices undertaken by most independent poultry farming households to make a living. In choosing to manage their farms in this way, farming households navigate the options that appear available to them that emerge as a result of factors such as the land and labour they have at their disposal.

Mixed farming systems do not inherently deviate from policies promoting biosecurity. It is thus not the existence of mixed farms that undermines biosecurity implementation in Viet Nam, but rather the practices farmers engage in to make a living through the combining of farming processes on these farms that will be discussed here in relation to Viet Nam’s biosecurity agenda.

Most farms in Viet Nam are operated by a household rather than an individual. External support is only brought in during very busy periods or for the few farms that decide to operate beyond the household’s capacity. As such, the labour that one has access to as part of the household is a key part in what is possible in terms of how farming activities are managed. Most participants across both contract and independent farms needed to distribute labour between the members of their household to maintain their farms, with chicken seen as an easy thing to raise alongside multiple other farming activities:

My family does other farming activities including orchards, rice farming, and aquaculture.… Raising chickens only takes up a part of the day, so I can maintain multiple jobs to increase the family’s income.’^20^

Mixed farming has ramifications for how farms are built and maintained, which can have consequences for how farmers can meet biosecurity requirements associated with poultry rearing. Among the farmers we spoke to, poultry production was most often their main source of income, but that does not mean the infrastructure on their farm was primarily developed with this in mind. Building chicken houses requires a lot of capital, so for many, their land was originally used for rice cultivation, orchards or growing vegetables, adding livestock only when they could afford it. Thus, for many poultry was introduced to their farms as an addition to other income generating activities. In some cases, several farming activities may be added over time and seen as working in symbiosis with one another, despite how differently they are managed and go on to be distributed through their respective networks. For example, someone with a poultry house may invest in a pond close by as a reliable way to cool it. Once they have the pond, farmers reported adding fish, ducks, or both as an additional income stream facilitated by that existing asset. In doing so, the pond may attract wild birds, and bring various species (whether wild birds, geese, or ducks) into close proximity to one another, potentially increasing the risk of disease incursions in their farm where, for one example, shared scavenging areas and mixing of poultry species have been associated with an increase in the risk from HPAI H5N1 outbreaks (Henning et al. 2009). While for veterinary practitioners these biosecurity concerns emerge as a priority, for farmers the investments made for poultry actually serve as a platform to easily sustain a diverse livestock portfolio. In doing so, farmers described being able to balance the income that can be generated from each activity across different seasons and during times they associate with greater risk:

In general, good husbandry should cause few diseases when keeping chickens, but keeping them, no one can avoid diseases entirely. In order to maintain the capital for raising chickens, one [activity] compensates for the other, sometimes when the money for chickens is not enough, it is taken from the money for cultivation and vice versa.’^21^

Despite describing regular and significant challenges, participants highlighted the centrality of chickens to their household income, with some even losing money or only managing to make small amounts through their diversified activities. Farmers justified their commitment to their diversified activities through their claims that this work provides much needed security. Research both from Viet Nam and beyond has repeatedly agreed with the farmers’ position on this, arguing that diversified livelihoods provides adaptability that makes households better able to sustain themselves and recovery from shocks (Suhardiman and Rigg 2021; Bui et al. 2013; Ellis 2000). For poultry farmers in Viet Nam, mixed farming might protect them a little when markets poultry prices are low, allow them to cease raising birds whenever they choose, and maintain options that they can turn to in the future if anything in the poultry sector changes and they decide to leave. Thus, while sustaining activities that are not as lucrative as poultry farming can appear to present biosecurity challenges or limit a farm’s income generating capacity, the security that maintaining a diverse portfolio of activities provides and the adaptability it allows for in how poultry are managed from one season to the next, makes mixed farming an indispensable feature of how many farmers choose to operate and sustain their livelihood.

### *Gà dù*

On many contract farms where raising chicken may be the only farming activity (although, not always), other points of disconnect with the national biosecurity agenda can arise as farmers respond to the different experiences, challenges and opportunities emerging from their relationship with (and outside of) the company they work with. Typically, contract farming involves an agreement between farmers and companies. These agreements usually include conditions informing the production of farm products, specify prices afforded to farmers, quantities and the quality of the product demanded by the contracting company, and the date for delivery to them. These agreements may also include details of responsibilities for farm management, oversight and input provision (FAO 2024). In Viet Nam’s poultry sector, contract farming adheres to this broad definition, with each company defining the specifics of these details with the farmers they work with to shape the details of what happens on the ground.

Several companies operate contract poultry farming contracts across Viet Nam. Local (e.g. Viet Tin Joint Stock Company and Viet Nam Green Feed) and international companies (e.g. Japfa and C.P Viet Nam) produce birds through contracted relationships with farms, providing inputs and varying levels of oversight to farmers in exchange for the mature birds which they then organize the slaughter or trade of. In return, farmers are guaranteed a minimum price from the sale of their birds as long as they reach a predefined minimum weight.

To establish a contract, farmers are required to pay an initial deposit based on the number of birds they commit to raising in each batch (usually about 5000 VND per bird). This deposit is returned to farmers if they end their contract and is only drawn from by the company if a farmer fails to return birds they have raised within their contracted agreement. Usually, companies accept that up to 5% of a batch of chicks may die, so they allow for that and expect at least 95% of birds to be returned at mature weight. All their calculations are based on this allowance, and the deposit is only used to compensate for additional unexpected losses. This deposit is a significant upfront cost, making contract farming inaccessible for many farmers. However, for those who can afford it, operating as a contract farm allows farming households to operate without paying for any inputs from the company upfront. Instead, these costs are deducted from what farms are ultimately paid when a batch of birds is collected.

Despite the guaranteed price offered, contracted farming households still regularly adopt livelihood strategies that extended their networks of operation and provided additional adaptability and income alongside the contract system. One such livelihood strategy is entirely unique to contract farms: stretching profits while maintaining contract relationships by raising birds referred to as *gà dù*. While some companies strictly condemn this practice, others turn a blind eye. Notably, this practice is recognised as against the rules by companies and farmers, meaning that the prominence of *gà dù* farming is hard to determine, with few wishing to talk about it openly.

Mrs Hoang provides a ready example of what this means for the operations of a contract farm. She has been contracted to Viet Tin for three years, and raisings 15–20,000 coloured hybrid birds per year. As part of her contract, she receives a minimum of 4000 chicks from the company per batch, but regularly opts to receive 6000 as her housing allows for this. She then sources an additional 500 chicks to keep alongside them—these are the *gà dù*.^22^ Once the birds have matured, while Mrs Hoang is obliged to sell 6000 back to the company (or at least 95% of them with allowance made for a number to die), she is free to manage the raising and sale of the additional 500 birds as she likes. Thus, after traders associated with the company have collected the relevant quantity of birds, Mrs Hoang contacts additional traders to negotiate the collection and sale of the remaining *gà dù*, just as an independent farmer would do.

As the additional birds sit outside of her contract agreement, Mrs Hoang does not receive inputs from the company for them, such as feed and medicines. Calculations for what the company provides is based solely on the 6000 she is committed to returning. Some farmers say they can stretch the feed and medicines the company provides to also raise the *gà dù*. This is a risky approach, however, as if birds do not reach a required weight, the company will charge farmers for the deficit, drawing from their deposit. Mrs Hoang prefers to buy additional feed and medicine from an external provider. This is preferable to buying more from the company as farmers say the feed is expensive relative to what they can get through local feed agents.

The same goes for medicines, which the company has an allowance for that farmers can claim as needed over the course of raising any batch. This will not cover the *gà dù*, so farmers source any additional medicines for these birds from external providers. This is something that contract farmers may opt to do for all their medicines anyway, as they receive additional pay from companies if they use less medicine than expected. Farmers said this additional pay was greater than the cost of medicine would be if they get cheaper medicines from local providers, rather than using the premium imported medicines many companies provide. This cost saving strategy both increases a contract farm’s potential profit, but also means the medicine provision and application across contract farms varies dramatically.

While *gà dù* never constitute a large portion of a farm’s earnings, by using their existing infrastructure and the provisions they receive as part of their contract farming operation, farmers say they can earn enough from *gà dù* to at least cover water, electricity and bedding costs, stretching the profit margins of their contract operation by negating some of the running costs of their farm that they would otherwise remain responsible for. They are also able to sell their birds’ waste as manure for fields, much as independent farmers do. This stretches the potential earnings from contract farming and enables farmers to benefit from the market when it is strong while still retaining the security of the agreement they have within the contract system. In doing so, contract farmers engage in a range of additional networks through which additional birds might be sourced, fed, medicated and sold.

This more versatile farming system challenges the biosecurity agenda accompanying the sectors growth as it complicates the relatively simple provision and onward sale business models that contract farms are presented as operating within—particularly by the large companies responsible for those contracts. The diversified sourcing and sale of *gà dù* may have consequences for pathogen incursions and other concerns such as antimicrobial use, as birds from various sources are brought together, sustained with feed and medicines acquired from various sources in whatever combinations make sense to the farmers, and are delivered and collected by both various company and independent traders for onward sale—where poorer quality or sick birds can also find a market where a contract company may otherwise have rejected them.

## Conclusion

Around the world, backyard, smallholder and independent commercial farmers operate alongside increasingly industrialised and contract agricultural and livestock production. Research from countries in both the global north and south has explored changing livestock and agricultural production systems that see international and national political and corporate agendas rub up against local lives and livelihoods generating both tensions and new practices (for example, from Bangladesh, Uganda and the USA see: Hinchliffe et al. 2021; Thompson 2021; Blanchette 2020). Intentionally or not, while rightly emphasising the challenges and constraints that workers and local producers are faced with in light of changing margins and policies, these works also reveal limits to the national and corporate powers that implicitly promote industrialised systems. For example, discussions on antibiotic use in livestock production increasingly recognises industrialisation as responsible for the increase in application, while also describing the ongoing challenges of limiting that use among farmers, regardless of the system, contract or country within which they operate (Hinchliffe et al. 2018). As Kayendeke et al. (2023) identified through their work on antibiotics and ‘quick farming’ in Uganda, the changes that intensification of livestock production and accompanying disease management brings reflect ‘a micro-biopolitical conundrum where the agendas of microbes, farmers, publics, authorities and transnational agencies are in tension’ (2023, p. 996). In South and Southeast Asia, one such tension associated with the growth of livestock sectors, intensification of land use and rapid urbanisation is the increase in zoonotic disease risks and how different organisations and individuals believe they should be managed (Ahmed et al. 2019; Blasdell et al. 2022; Horby et al. 2014).

Concerns surrounding zoonotic diseases in global health communities and their overlap with food safety and national productivity has contributed to the creation of biosecurity narratives that are circulated in various ways at international and national levels. In some contexts, such as in Viet Nam, biosecurity is seen as a vital part of the country’s growth and export agenda, in turn shaping the kind of biosecurity policies that are ultimately introduced. As Hinchliffe et al. (2013) have identified, these biosecurity narratives often situate disease in space and encourage biocontainment and bioexclusion practices that hinge on borders around risky or at-risk species—prioritising certain lives and ways of living over others in doing so. It is with these borders and the associated rigidity of disease surveillance and response guidelines that make up much of biosecurity guidance, that we can begin to identify challenges for local populations responsible for implementation. A livelihoods approach provides a starting point to explore these challenges from, with a long history of research that acknowledges how livelihoods are often made resilient through adaptability and diversification—two things that are fundamentally at odds with the increasingly sterile, static and bounded forms of biosecurity promoted in industrialised livestock. It is because of this disconnect between local practices and global health perspectives that researchers, the transnational health community and local governments need to understand how actors within livestock production and distribution networks are making a living and why. As a start to building this understanding, we have described key factors shaping coloured chicken farming practices in Viet Nam, including motivations, breeds, seasons, and income securing activities. Relating to these factors, we have explained their connection to a household’s efforts to stabilise or enhance their livelihoods, while also reflecting on how they necessitate the avoidance or undermining of a national biosecurity agenda.

The flexibility to judge when to raise birds, in what quantities, and across how many houses and batches all emerged as factors informing how poultry farming households are making a living. Participants described the need to make judgements about these details, which arose partly in response to changing weather, markets and disease risks across the year. In addition, participants describe the constraining factors influencing how their household managed their farm, including the number of members in the household who could contribute, their liquid assets (or credit capacity), and the land they had access to. This has resulted in practices that specifically facilitate this adaptability, such as staggered batch management. Frequent changes in scale and between specific relationships within the sector (and beyond) provides resilience to farmers, but can also lead to potential disease risks or create environments that may limit the efficacy of the disease management that is being applied. For example, the staggered system of raising and selling birds can limit the cleanliness of houses and create a greater change of transmission between infected birds within a farm as birds from multiple batches overlap and come into contact with one another or the detritus another batch produces in the farming environ. In doing so, even a farmer who works hard applying vaccines or cleaning poultry houses, may find their efforts undermined with a greater number of trading vehicles entering their farm or with houses spending less time empty between batches. Farmers are thus not intentionally rejecting the government’s or a contracting company’s biosecurity advice when they implement particular farming practices, but rather balance their experience and the needs of their farm against the advice to ultimately select and apply biosecurity measures and alternative disease management strategies (such as ceasing poultry farming activities at certain times) which can work alongside the other livelihood strategies and relationships they seek to maintain.

Looking to the future, the Vietnamese government’s commitment to larger more industrialised farming operations is influencing land use, international company interest, and ongoing development in the poultry sector (among others). Contract farms have emerged as a result of both local and international company arrangements within the sector and appear to align with the government’s ambition to continue to sustain the growth of the sector while seeking to improve Viet Nam’s biosecurity measures and food safety standards. However, our research indicates that greater diversity exists within the infrastructures, operations and networks within which contract farms in Viet Nam currently operate than is often thought. For those on contract farms, adaptability from season to season is less relevant as they receive a stable price across the year. However, navigating seasonal disease concerns, combined with the appeal of the sector as an easy and profitable business to engage in influences how people choose to run their farms, which vary more than advocates of integrated contracted farming systems would like. The raising of *gà dù* presents just one way that farming household are able to adapt their operations in light of contracts but maintain some level of flexibility and connection to extended networks of other traders and markets within the sector. Thus, consideration should be given to what the scaling up of any particular single type of farm or intervention could mean for both disease risk management and the livelihoods of those expected to implement or abide by the changes.

Overall, the different farming practices present within Viet Nam’s poultry sector provide an example of the complexities present in a larger interconnected livestock and agriculture system. While seeking solutions for complex transnational health problems, it is important to keep sight of the local actors and diverse livelihood strategies available in different contexts as they will ultimately be impacted by (and impact upon) any efforts to address them. Acknowledging context and networks of actors engaged in various livelihood activities challenges existing policy approaches and the popular One Health paradigm to think of interventions as something that goes beyond a single disease, individual or farm and thus consider solutions that are both considerate of the systems on which people depend to sustain a living, but also understand where systems and power lie that are ultimately shaping local practices.

## Footnotes

1 Coloured chickens is a grouping used here to refer to the indigenous and hybrid broiler breeds that dominate the local market. They are discernible from their red, black or brown feathers,, as opposed to the white broiler breeds that dominate the sector in many other contexts.

2 *Gà dù* is the term used to refer to additional coloured chickens raised alongside those being raised within a contract farm. Sourced separately but raised together, these birds fall outside the management of contract companies but can benefit from company provisions, allowing farmers to engage in local markets alongside their regular contracted farming activities.

3 Table 1 provides a summary of the full range of farms visited including those with fewer birds per batch and white broilers that informed our understanding but are not discussed directly in this study.

4 Along with zoonotic diseases such as avian influenza (H5N1) and swine flu (H1N1), epizootics such as bovine tuberculosis and foot and mouth disease have also greatly contributed to the shape of biosecurity narratives in recent decades (see Hinchliffe et al. 2013).

5 For example, the government has been investing in large industrial slaughtering facilities, and sought to limit market-based slaughtering of live birds, particularly in urban centres (Vu and Ebata 2024).

6 Here non-industrial is inclusive of the large commercial farms on which this study focuses.

7 Interview, independent poultry farmer, Ha Tay district, Hanoi.

8 Pigs have dropped to become the second largest livestock sector in Viet Nam following a decrease in production due to an outbreak of African Swine Fever in 2019 (Nguyen-Thi et al. 2021). Like poultry, in recent years, the Vietnamese government has sought to expand the sector but unlike poultry they come with serious environmental risks through waste. Thus, the government has introduce measures to limit the environmental impact from waste, including incentives for farmers to invest in biogas digesters. These digesters are now a common feature of commercial pig farms, with money and land committed to this widely accepted process. While enforcement of rules surrounding waste management is limited, farmers are aware of the expectation surrounding waste management, with most commercial producers (80%) investing in the infrastructure needed (Dinh 2017). For farmers such as Mr Luu the perceived additional constraints and investments associated with how waste is managed and the farm’s environmental impact influence decisions to rear poultry. Poultry comes with no such requirements, saves land for other purposes and waste can be sold for additional profits.

9 With growth in production, import and consumption of meat in Viet Nam, how birds are bought and consumed is diversifying. Both local and imported white broiler chicken meat is readily available in the growing number of supermarkets and convenience stores in Viet Nam’s cities. While a shift in acceptance of chilled meat is certainly growing (Hoang et al. 2023; Hansen and Jakobsen 2020), wet markets selling live birds are still the primary source of chicken in Viet Nam. The markets sell both live birds, fish and chilled pork and poultry meat depending on the area and infrastructure.

10 Maturity is a term here that refers entirely to a bird’s saleable weight. Age is primarily used to estimate the amount of time it takes for a bird to reach this weight and the amount of food that they will require within that time.

11 Interview, independent poultry farmer, Ha Tay district, Hanoi.

12 Animal welfare (*phúc lợi động vật*) was not a concept that was explicitly mentioned by any of the farmers we met with and on the whole Viet Nam has been pointed to in international animal welfare communities as lacking in sufficient protections for animals (Eurogroup for Animals 2022). Despite this, not mentioning welfare does not mean acts of care were not implicit to farming activities for many farmers e.g. providing outdoor space and allowing birds to explore hills or perch in trees.

13 *Gà xấu* or ‘ugly chicken’ was a phrase associated with birds that appeared less attractive than others. It is not a reflection of specific sickness or disease, so much as appearance or behaviour based judgements used to categorize birds and their prices. That does not exclude diseased birds from inclusion in this category, with no distinction made in the markets. This is more likely to happen at the farm level, with diseased birds and poor-quality birds being sold with the help of specific traders.

14 Interview, independent poultry farmer, Hai Duong.

15 Interview, independent farmer, Hai Duong.

16 Tết is a national holiday celebrating Vietnamese New Year. It takes place in late January or February each year in line with a lunar calendar date system.

17 Interview, independent poultry farmer, Gia Lâm district, Hanoi.

18 Lunar calendars following moon phases to indicate the start of each calendar month. Here January and February on the lunar calendar align with February and March on the Gregorian calendar.

19 Interview, independent poultry farmer, Ha Tay district, Hanoi.

20 Interview, contracted poultry farmer, Chương Mỹ District, Hanoi.

21 Interview, independent poultry farmer, Hai Duong.

22 The extra 500 can come from contracted companies who turn a blind eye and are happy for farmers to say they had excessive deaths in the batch to cover the deficits that are returned. More often, these additional chicks come from external hatcheries.
